# Editorial: Epigenetics in cancer: mechanisms and drug development-volume II

**DOI:** 10.3389/fgene.2023.1242960

**Published:** 2023-07-05

**Authors:** Xiao Zhu, Zhenhua Xu, Biaoru Li

**Affiliations:** ^1^ Southern Marine Science and Engineering Guangdong Laboratory (Zhanjiang), Guangdong Medical University, Zhanjiang, China; ^2^ Zhejiang Provincial People’s Hospital, People’s Hospital of Hangzhou Medical College, Hangzhou Medical College, Hangzhou, China; ^3^ Center for Cancer and Immunology, Children’s National Health System, Washington, DC, United States; ^4^ Cancer Center, Medical College of Georgia, Augusta University, Augusta, GA, United States

**Keywords:** editorial, epigenetics, cancer, DNA methylation, RNA methylation

Cancer remains the leading cause of death in affluent nations, underscoring the urgent need for innovative approaches to combat this devastating disease (Siegel et al., 2023). Epigenetics, a field of study focusing on the modulation of gene expression and function without altering the DNA sequence, holds tremendous promise in understanding and addressing cancer development and progression.

The Research Topic titled “*Epigenetics in Cancer: Mechanisms and Drug Development-volume II*” presents a compilation of ten articles contributed by over sixty esteemed authors in the fields of cancer epigenetics and therapeutics. This comprehensive collection encompasses diverse research directions, including the roles of transcription and chromatin in gene regulation, DNA modifications, RNA epigenetics, non-coding RNA, and epigenomic methods. Alongside shedding light on the latest discoveries regarding epigenetic mechanisms, these articles also emphasize novel and promising therapeutic drugs aimed at reversing specific epigenetic alterations.

DNA methylation, a key epigenetic modification, has been extensively studied due to its involvement in transcriptional inhibition and gene silencing (Liang et al., 2021). Recent investigations have elucidated the downstream mechanisms of gene silencing mediated by DNA methylation, uncovering the remarkable contribution of molecular domain proteins (Wurster et al., 2021). In hepatocellular carcinoma (HCC), the understanding of epigenetic abnormalities linked to aberrant enhancers provides novel insights into drug therapy for this malignancy. It has been observed that DNA methylation can finely tune gene expression by balancing the effects of transcriptional inhibition and activation, highlighting its role in gene repression (Goncharova et al., 2023).

For instance, Yang et al. demonstrated the involvement of promoter methylation and miR-454-3p in the dysregulation of 4.1N/EPB41L1 at the transcriptional and posttranscriptional levels, respectively. These findings support the potential therapeutic use of targeting DNA methylation and miR-454-3p for NSCLC treatment. Additionally, Huang et al. analyzed epigenomic and transcriptomic data, proposing a prognostic signature based on six AE-DEGs that outperforms previous models in predicting long-term and short-term overall survival in HCC patients. Their discovery of the unique role of epigenetic aberration-induced aberrant enhancers in HCC progression offers new insights for drug therapy.

RNA methylation, another significant epigenetic modification, has emerged as a major focus of research. With over 100 chemical modification methods identified, N6-methyladenine (m6A) stands out as a predominant RNA modification (Zhou et al., 2020). Studies have highlighted the reversible nature of m6A modification, controlled by writers, readers, and demethylases. M6A plays a critical role in regulating gene expression, splicing, RNA editing, RNA stability, and controlling mRNA lifetime and degradation (Li et al., 2023; Xiong et al., 2023). Notably, the clinical and prognostic value of m6A-related features has been elucidated in glioblastoma multiforme (GBM), laying a foundation for future research in glioma (Liu et al.). Furthermore, researchers have emphasized the potential of ncRNA m6A modification and m6A regulators as promising diagnostic and prognostic biomarkers across various cancers, aiding in recurrence and survival prediction, and serving as potential therapeutic targets in cancer treatment (Chen et al. and Mobet et al.). However, while these advancements offer significant promise, further exploration is necessary to unravel more specific mechanisms and develop theories closer to practical applications in clinical diagnosis and treatment.

Another crucial post-transcriptional modification, 5-methylcytosine (m5C), has demonstrated a pivotal role in gene expression and RNA stability. In hepatocellular HCC, the characterization of m5C-related regulators has enhanced our understanding of the tumor immune landscape and provides a practical tool for predicting prognosis (Liu et al.). This valuable insight has the potential to improve patient outcomes and guide effective interventions for this challenging disease.

Although epigenetic modifiers have shown promise as targets for cancer treatment, their efficacy as standalone therapies remain limited. Combinatorial approaches that integrate epigenetic therapies with other anti-tumor treatments offer a more comprehensive strategy for maximizing therapeutic outcomes (Ye et al., 2021; Li et al., 2022; Lin et al., 2023).

In conclusion, the study of epigenetics in cancer has unveiled intricate mechanisms and opened new avenues for drug development. This collection of articles provides a snapshot of the latest research, encompassing diverse aspects of epigenetic regulation in cancer. As scientists delve deeper into these mechanisms and translate their findings into clinical practice, we anticipate further breakthroughs that will transform the landscape of cancer treatment and improve patient outcomes.
